# Effect of mixed light emitting diode spectrum on antioxidants content and antioxidant activity of red lettuce grown in a closed soilless system

**DOI:** 10.1186/s12870-023-04364-y

**Published:** 2023-07-06

**Authors:** Sopanat Sawatdee, Teeraya Jarunglumlert, Prasert Pavasant, Yasuko Sakihama, Adrian E. Flood, Chattip Prommuak

**Affiliations:** 1 School of Energy Science and Engineering, Vidyasirimedhi Institute of Science and Technology, Wang Chan, Rayong, 21210 Thailand; 2 Faculty of Science, Energy and Environment, King Mongkut’s University of Technology North Bangkok (Rayong Campus), Ban Khai, Rayong, 21180 Thailand; 3 Tree Moments Co. Ltd, Bangrak, Bangkok, 10500 Thailand; 4 Graduate School/Research Faculty of Agriculture, Hokkaido University, Sapporo, 060-8589 Japan; 5 Energy Research Institute, Chulalongkorn University, Pathumwan, Bangkok, 10330 Thailand

**Keywords:** Phenolics productivity, Antioxidant enzymes, Molecular antioxidants, Light spectrum, Continuous light, Hydroponic cultivation

## Abstract

**Background:**

Light spectra have been demonstrated to result in different levels of comfort or stress, which affect plant growth and the availability of health-promoting compounds in ways that sometimes contradict one another. To determine the optimal light conditions, it is necessary to weigh the vegetable’s mass against the amount of nutrients it contains, as vegetables tend to grow poorly in environments where nutrient synthesis is optimal. This study investigates the effects of varying light conditions on the growth of red lettuce and its occurring nutrients in terms of productivities, which were determined by multiplying the total weight of the harvested vegetables by their nutrient content, particularly phenolics. Three different light-emitting diode (LED) spectral mixes, including blue, green, and red, which were all supplemented by white, denoted as BW, GW, and RW, respectively, as well as the standard white as the control, were equipped in grow tents with soilless cultivation systems for such purposes.

**Results:**

Results demonstrated that the biomass and fiber content did not differ substantially across treatments. This could be due to the use of a modest amount of broad-spectrum white LEDs, which could help retain the lettuce’s core qualities. However, the concentrations of total phenolics and antioxidant capacity in lettuce grown with the BW treatment were the highest (1.3 and 1.4-fold higher than those obtained from the control, respectively), with chlorogenic acid accumulation (8.4 ± 1.5 mg g^− 1^ DW) being particularly notable. Meanwhile, the study observed a high glutathione reductase (GR) activity in the plant achieved from the RW treatment, which in this study was deemed the poorest treatment in terms of phenolics accumulation.

**Conclusion:**

In this study, the BW treatment provided the most efficient mixed light spectrum to stimulate phenolics productivity in red lettuce without a significant detrimental effect on other key properties.

**Supplementary Information:**

The online version contains supplementary material available at 10.1186/s12870-023-04364-y.

## Background

Red leaf lettuce, *Lactuca sativa* L., has become an important leafy salad vegetable that is consumed all over the world [1]. The increase in popularity of lettuce is partly because it is an excellent source of natural health-promoting antioxidants such as phenolic acids, flavonoids, and anthocyanins [2]. Epidemiological studies have shown that the phenolic compounds contained in vegetables can reduce the risk of developing cancer, as well as neurodegenerative and cardiovascular diseases [2, 3]. The health benefits of the consumption of natural antioxidants have brought about a considerable increase in the number of consumers who consume plant-based foods [4]. Recently, increasing the amount of these compounds in fruit and vegetables has been a focus of research. Among agricultural factors, light is an essential input that affects not only photosynthesis as a primary metabolism. Moreover, varying light conditions can lead to different degrees of comfort or stress, which influence the synthesis of second metabolites such as types of antioxidants, including anthocyanins, carotenoids, or flavonoids [5, 6]. Differences in photoperiod, intensity, and wavelength of light cause changes in the expression of a large number of specific plant genes, which show complicated responses that are difficult to predict [7, 8]. Consequently, various studies have utilized artificial light technology to study the effect of light on plant growth. Currently, the use of light-emitting diodes (LEDs) has expanded in horticultural applications [9]. Although the wavelength of LEDs depends on the type of semiconductor materials, LEDs can produce narrow-band spectra that can be selected to match plant photoreceptors and directly influence plant development [8]. Moreover, LEDs can provide several benefits, such as safety, lower heat emission, easily controlled light output, and decreased electricity consumption [10–12]. Red and blue LEDs have been reported to have positive effects on plant growth and nutritional development. For instance, the biomass production of lettuce cultivars was increased under 660–690 nm red LEDs [13], whereas 640 nm red LEDs did not increase lettuce growth but activated its antioxidant system [14–17]. Applications of blue LEDs (400–500 nm), alone or in combination with red LEDs, have been found to possess positive impacts on plant growth and secondary metabolites. For instance, past research revealed an increase in biomass [18, 19], vitamin C [18], carotenoids [15, 20], flavonoids [21], total phenolics [22] and level of pigmentation [23] in both green and red lettuces. Modified lighting was established to improve the properties of the plants in response to the demands of consumers concerned about the nutrition of foods [21]. However, some studies reported that different model plants responded differently to specific light spectra [24–26]. This means that changes in plant growth caused by light also depend on plant species [10]. Moreover, works concerning light wavelengths focused primarily on blue and red, while the influence of green was vague [27–29]. Green light (500–600 nm) has either been found to be inactive for plant growth in certain species such as peppers, wheat, cucumbers, and soybeans [30, 31] or has led to a reduction in lettuce mass production [32]. Despite the fact that green light is reflected from the plant surface, this particular wavelength is somehow capable of penetrating into the plant canopy more effectively than blue and red, thereby facilitating photosynthesis [33]. Past research revealed that the application of green light in cultivation also demonstrated positive effects such as promoting lettuce growth and its antioxidant capacity [33–35].

In general, plants grown under stress can produce high levels of antioxidants [36, 37] as their antioxidant defense systems (non-enzymatic and enzymatic) are activated to eliminate or delay oxidative stress caused by the overproduction of reactive oxygen species (ROS). The main enzymatic antioxidants are superoxide dismutase (SOD), catalase (CAT), ascorbate peroxidase (APX), and glutathione reductase (GR) [38–40]. SOD, as the first line of defense, will remove O_2_^•-^ by catalyzing its dismutation [39]. Subsequently, CAT and APX are both important for eliminating H_2_O_2_ and other hydroperoxides [41]. In addition, GR is necessary for activating forms of antioxidants in the ascorbate-glutathione cycle [42]. However, if stress conditions lead to excess ROS, plants are damaged, and cell death eventually occurs [39, 43]. As a result, finding optimal growing conditions to produce antioxidant-rich vegetables has been challenging. Although numerous works [44–47] showed the effect of light type on plant growth and antioxidant defense system, little information on the simultaneous study of anti-oxidative enzyme activities, especially those mentioned above, and compounds as defensive mechanisms of lettuce grown under various light wavelengths is reported.

The aim of this research was to examine the effect of continuous LED spectra including white (W, control), blue supplemented with white (BW, λ_peak_ 442 nm), green supplemented with white (GW, λ_peak_ 517 nm) and red supplemented with white (RW, λ_peak_ 630 nm) on red lettuce’s enzymatic and non-enzymatic antioxidants, which are excellent natural health-promoting compounds. Herein, a soilless cultivation system was carried out in order to provide more efficient nutrient management and avoid soil pollution. It is quite common to measure the concentration of antioxidant compounds in terms of the amount of compounds per unit mass as some treatments may appear promising in terms of antioxidant concentrations [35, 48–52]. However, biomass yield (vegetable weight per planting area per unit of growth time) can sometimes show a negative correlation with antioxidant accumulation [53]. In this regard, it is important to also consider their productivity, which is the amount of antioxidant compounds produced per planting area per unit of growth time. Therefore, this study will provide clear tradeoff between biomass yield and phenolics productivity.

## Results

In the current study, the treatment of the red lettuce with pure white LEDs, denoted as W, indicates the control. BW, RW, and GW signify the grow light setups in which blue, red, and green were, respectively, supplemented with white.

### Fundamental properties (plant growth, fiber content and appearance)

**Fig. 1:**
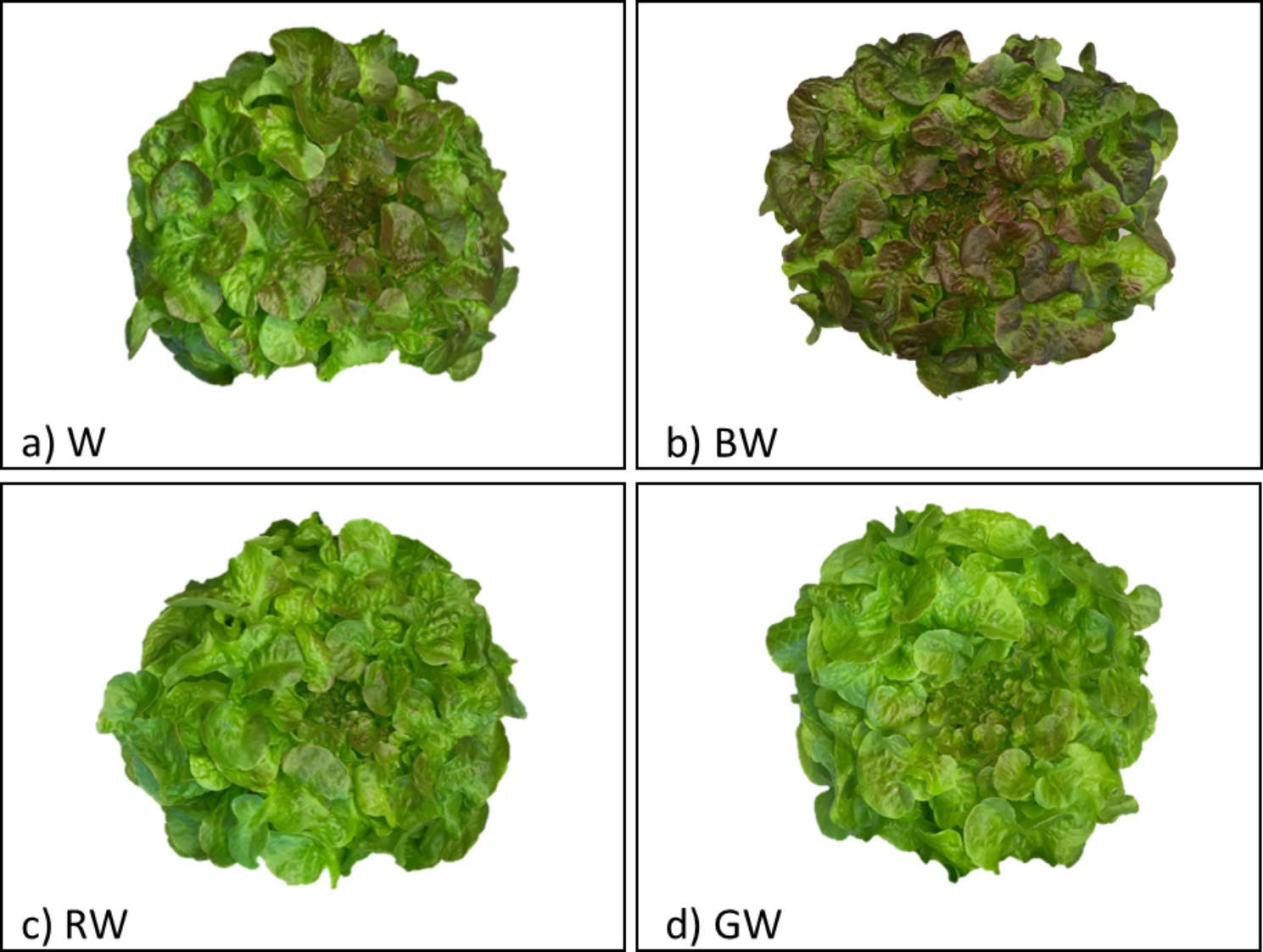
Morphology of red lettuce at Day 45 of cultivation grown under different spectra with 105 ± 10 µmol⋅m^− 2^⋅s^− 1^ total intensity for all treatments; (**a**) W (white), (**b**) BW (blue supplemented with white, λ_peak_ 442 nm), (**c**) RW (red supplemented with white, λ_peak_ 630 nm) and (d) GW (green supplemented with white, λ_peak_ 517 nm)

Lettuce characteristics including appearance, fresh weight and dietary fiber content were measured in the current study. These characteristics were reported on Day 45 of cultivation, which is the final growth stage of the mature red lettuce from indoor agriculture. The effect of the continuous LED spectra on the morphological appearance of the lettuce is shown in Fig. 1. It can be seen clearly that red lettuce leaves grown under the BW treatment (Fig. 1b) had a stronger red appearance than when using white light. The RW and GW treatments both produced lettuce with mostly bright green leaves, with features shown in Fig. 1c and d.

**Fig. 2:**
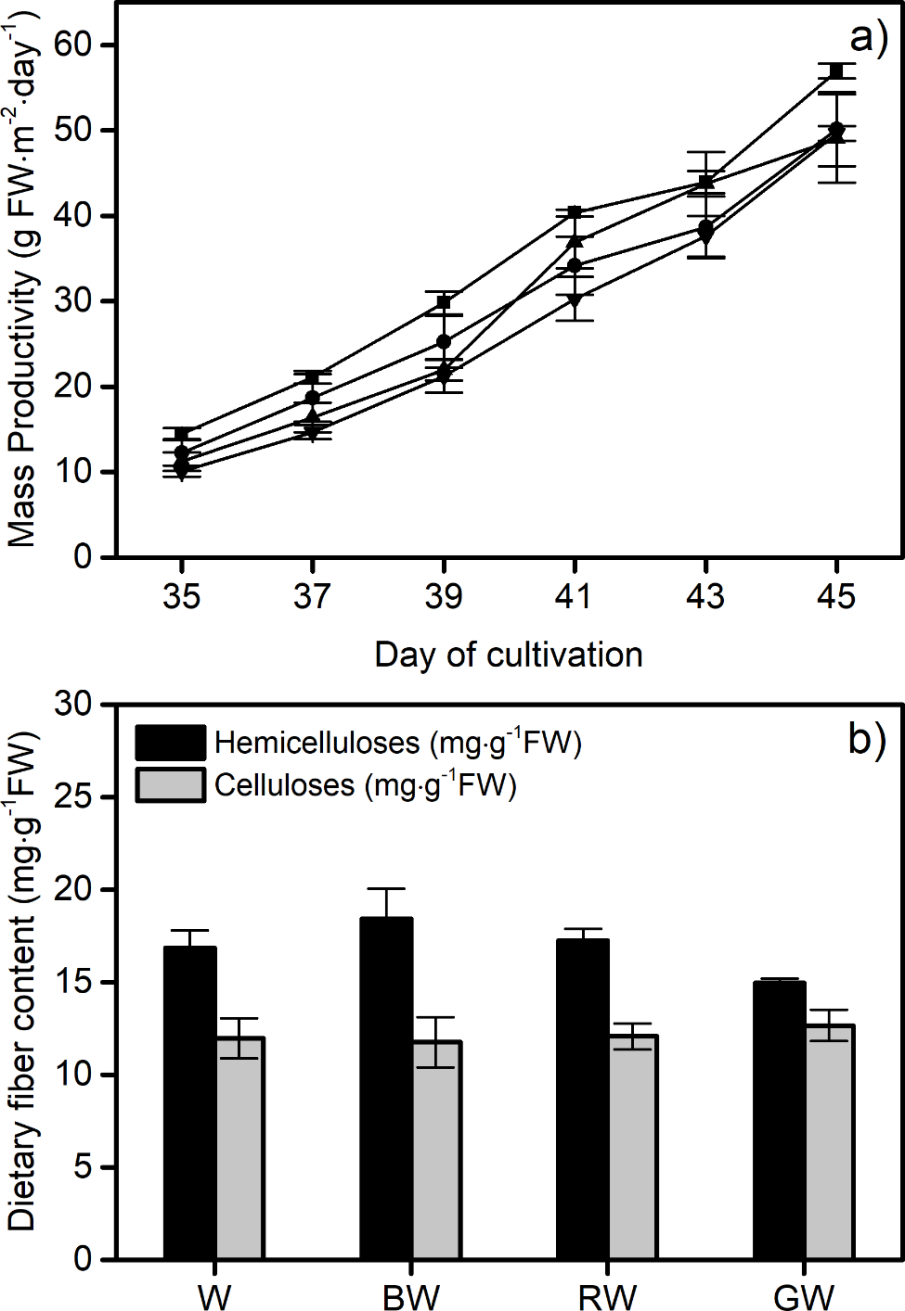
Fundamental properties including (**a**) mass productivity of white (■), BW (blue supplemented with white, λ_peak_ 442 nm, ●), RW (red supplemented with white, λ_peak_ 630 nm, ▲) and GW (green supplemented with white, λ_peak_ 517 nm, ▼) and (**b**) dietary fiber content of red lettuce grown under different spectra. Vertical bars represent mean ± standard error

Edible fresh weight is one of the main quality factors for any crop. This study also demonstrates the influence of different spectra of LEDs on the mass productivity of lettuce, as shown in Fig. 2a. The fresh weight of lettuce was measured to determine biomass productivity (gram of FW per unit of planting area per unit of time). The current study indicates that different LED spectra did not have a significant impact on the mass productivity of red lettuce, which was found to be in the range of 56.9 ± 1 to 49.1 ± 5 g FW⋅m^-2^⋅day^-1^ at the final stage of growth. As shown in Fig. 2b, differences in LED spectra in cultivation had a non-statistically significant effect on either the hemicellulose or the cellulose content, which ranged from 14.9 ± 0.4 to 18.4 ± 3 mg⋅g^-1^ FW and 11.8 ± 2 to 12.6 ± 1 mg⋅g^-1^ FW, respectively.

### Phenolic compounds accumulation

**Fig. 3:**
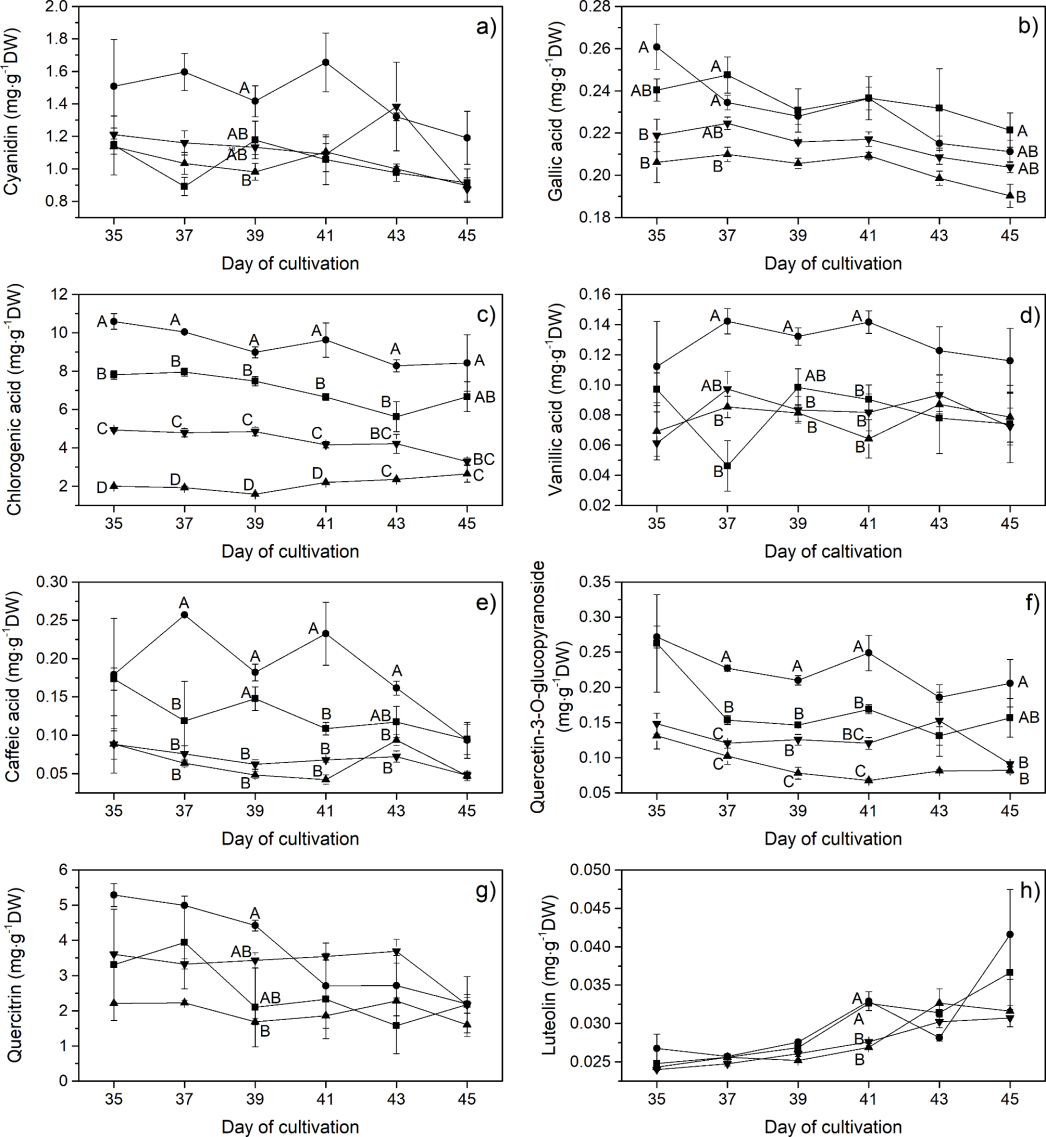
Individual specific phenolic compounds (mg⋅mg^− 1^DW) of red lettuce grown under different light spectra including W (white, ■), BW (blue supplemented with white, λ_peak_ 442 nm, ●), RW (red supplemented with white, λ_peak_ 630 nm, ▲) and GW (green supplemented with white, λ_peak_ 517 nm, ▼) measured via HPLC. Vertical bars represent mean ± standard error. The capital letters represent statistical significance on analyzed day (Day 35 to 45) between different LED spectra. The absence of a letter label indicates no statistical significance between different LED spectra

In the current study, the accumulations of phenolic compounds were measured on the 35th, 37th, 39th, 41st, 43rd, and 45th days of cultivation to study changes in the non-enzymatic antioxidant defense system in response to continuous light with different spectra. The results show that each LED spectrum significantly influenced the phenolic content of lettuce grown under closed soilless cultivation. HPLC was used to determine the accumulation of individual phenolic compounds during lettuce cultivation, including cyanidin-3-glucoside, gallic acid, chlorogenic acid, vanillic acid, caffeic acid, quercetin-3-O-glucopyranoside, quercitrin and luteolin (Fig. 3). The data indicates that the BW treatment significantly promoted the production of four major phenolics, including gallic acid, chlorogenic acid, quercetin-3-O-glucopyranoside, and vanillic acid. The amounts of these compounds were found to be 11.0%, 217.7%, 150.9%, and 47.5%, respectively, higher than those obtained from the RW treatment. Interestingly, only luteolin accumulation in all treatments showed a significant increase from Day 35 till Day 45 of cultivation; it increased 47.6% in the control, 55.3% under the BW spectrum, 30.0% under the RW spectrum, and 27.9% under the GW spectrum over this period. Apart from gallic acid, chlorogenic acid, and quercetin-3-O-glucopyranoside, most phenolic compounds at Day 45 were not statistically significantly influenced by the light spectrum used, as shown in Fig. 3a-h.

**Fig. 4:**
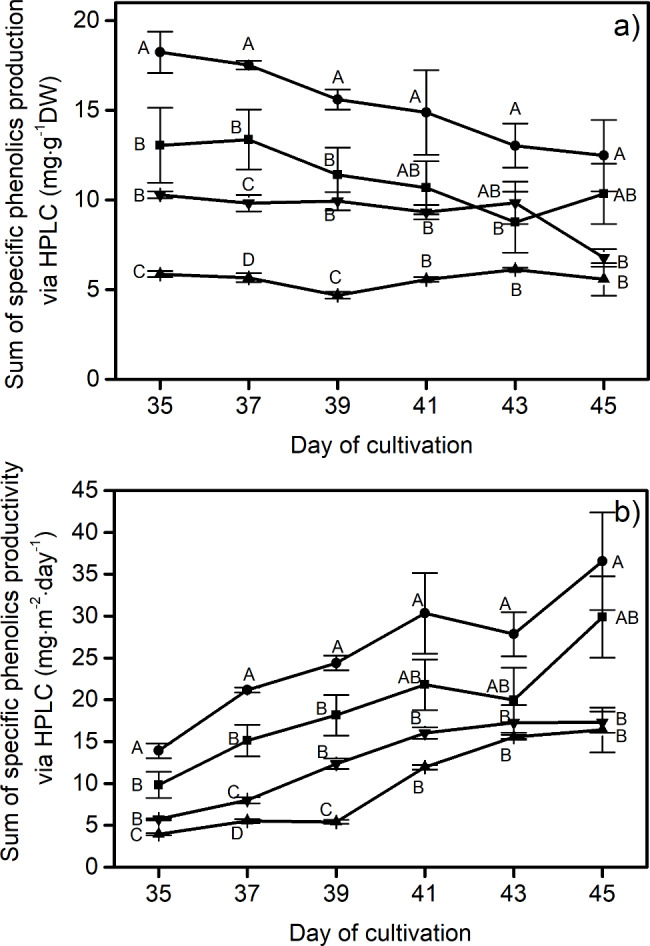
Summation of specific phenolic compounds via HPLC measurement including (**a**) specific phenolics production and (**b**) specific phenolics productivity of red lettuce grown under different light spectra including W (white, ■), BW (blue supplemented with white, λ_peak_ 442 nm, ●), RW (red supplemented with white, λ_peak_ 630 nm, ▲) and GW (green supplemented with white, λ_peak_ 517 nm, ▼). Vertical bars represent mean ± standard error. The capital letters represent statistical significance on analyzed Day (day 35 to 45) between different LED spectra

In the overall picture, Fig. 4a demonstrates the summations of these eight phenolic contents, which were found highest in the lettuce aged 35 to 39 days that underwent the BW treatment. In particular, on the 35th day of cultivation with the BW, the control, and the GW treatments, the sums of the eight phenolics were 18.2 ± 1 mg⋅g^-1^ DW, 13.0 ± 2 mg⋅g^-1^ DW, and 10.3 ± 0.2 mg⋅g^-1^ DW respectively. In contrast, low production of these phenolics of around 5.9 ± 0.2 mg⋅g^-1^ DW was found from lettuce grown under the RW spectrum. In terms of phenolics productivity, which was calculated by multiplying dry mass productivity (g DW⋅m^-2^⋅day^-1^) by the sum of phenolics production per g DW, 45-day lettuce grown under BW spectra yielded the eight phenolics with comparable productivity (36.5 ± 5.8 mg⋅m^-2^⋅day^-1^) to the white treatment (29.9 ± 4.8 mg⋅m^-2^⋅day^-1^), as shown in Fig. 4b. It also indicates that the changes in phenolic productivity with respect to time of GW and RW treatment were under 20 mg⋅m^-2^⋅day^-1^ until the end of the growth period: 17.3 ± 1 for GW and 16.4 ± 2 for RW at Day 45.

**Fig. 5:**
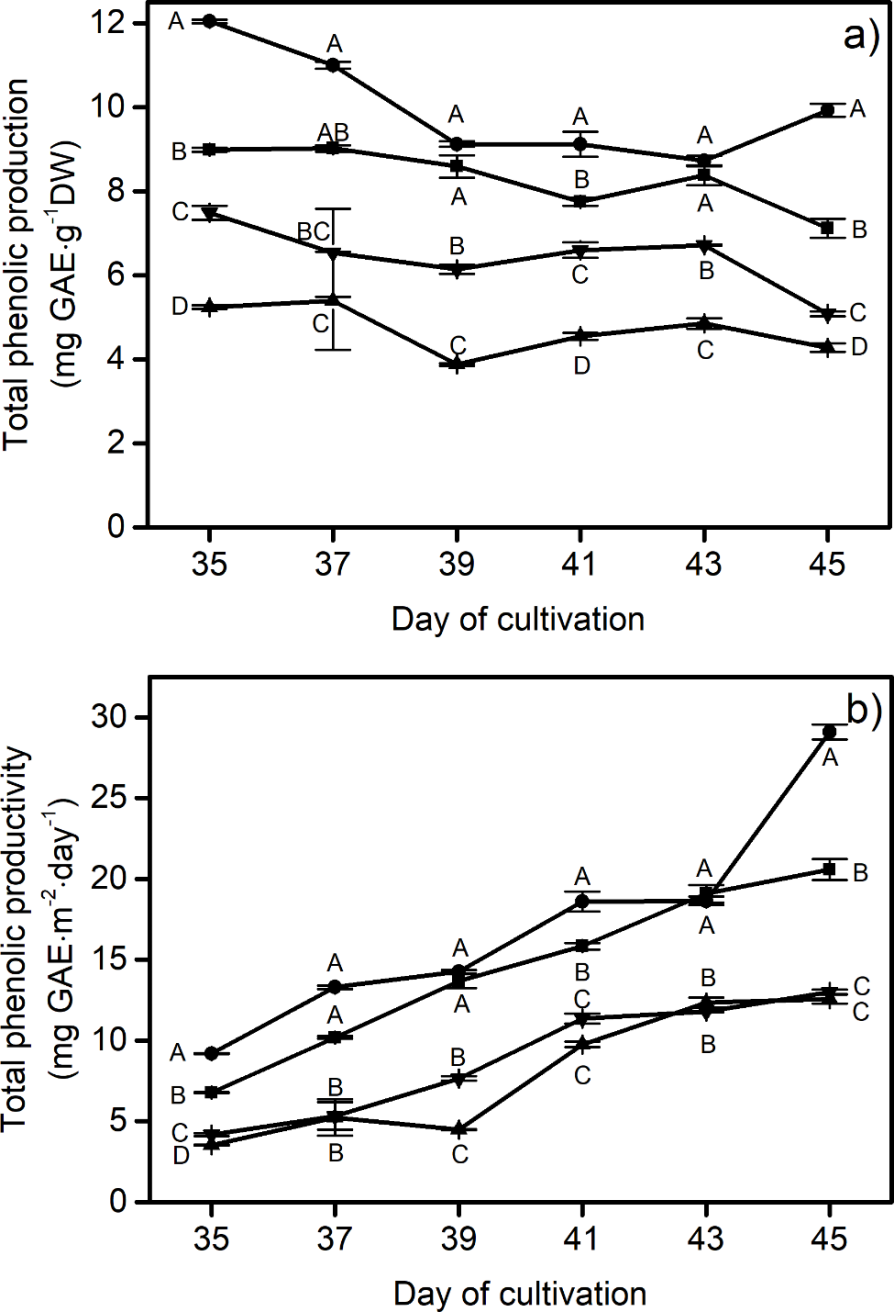
Folin-Ciocalteu measurement of total phenolic content including (**a**) total phenolic production and (**b**) total phenolic productivity of red lettuce grown under different light spectra including W (white, ■), BW (blue supplemented with white, λ_peak_ 442 nm, ●), RW (red supplemented with white, λ_peak_ 630 nm, ▲) and GW (green supplemented with white, λ_peak_ 517 nm, ▼). Vertical bars represent mean ± standard error. The capital letters represent statistical significance on analyzed day (Day 35 to 45) between different LED spectra

Moreover, the Folin-Ciocalteu method was applied to confirm the accumulation of total phenolics. The results show similar trends to those obtained from the HPLC. Figure 5a shows that the total phenolics accumulation for most treatments (mg GAE⋅g^-1^ DW) slightly decreased throughout the time of measurement, except for the RW treatment. Overall, the BW treatment was observed to produce lettuce with higher amounts of total phenolic compounds, ranging from 9.1 ± 0.1 to 12.04 ± 0.04 mg GAE⋅g^-1^ DW, compared to those from the W, GW and RW, all of which yielded under 10 mg GAE⋅g^-1^ DW. At the end of the growth period (Day 45), the BW treatment produced 39.5%, 95.1% and 132.0% higher amounts of total phenolics than the W, GW and RW treatments, respectively. Total phenolics productivity, or the amount of phenolics produced per unit of planting area per day of growth, was investigated as a tradeoff between mass productivity and bioactive component production. The results show that in most of the measurements, the highest total phenolics productivity (Fig. 5b) was found under the BW treatment, in the range of 9.2 ± 0.1 to 29.1 ± 0.5 mg GAE⋅m^-2^⋅day^-1^, which were approximately 41.4% higher than that under the control treatment at the final stage. On Day 45, RW and GW treatments provided the same total phenolics productivities, approximately 13 mg GAE⋅m^-2^⋅day^-1^.

### Total antioxidant activity

**Fig. 6:**
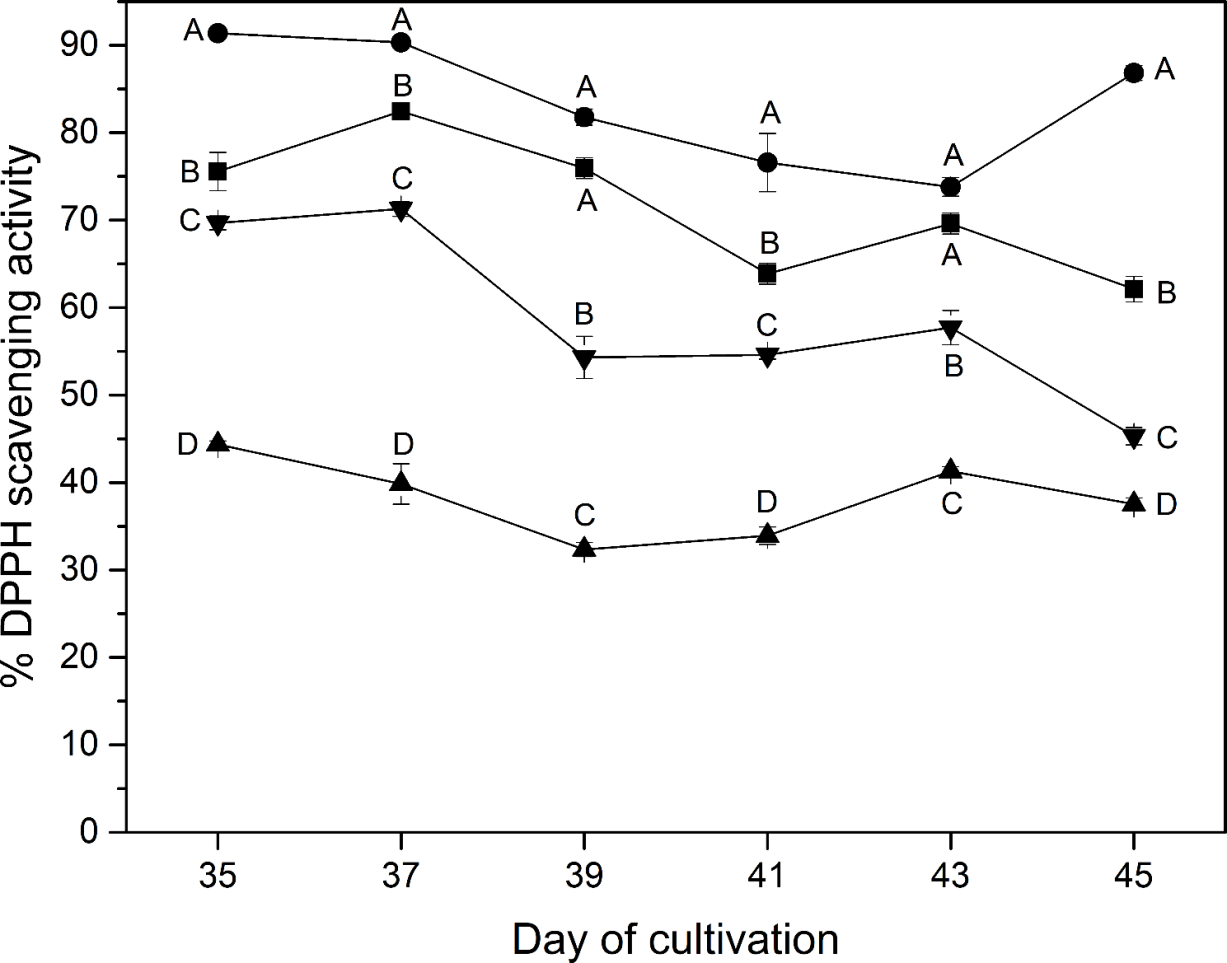
Antioxidant activity (percentage of DPPH free-radical scavenging) of 10 mg DW⋅mL^− 1^ methanolic lettuce extraction under different light spectra including W (white, ■), BW (blue supplemented with white, λ_peak_ 442 nm, ●), RW (red supplemented with white, λ_peak_ 630 nm, ▲) and GW (green supplemented with white, λ_peak_ 517 nm, ▼). Vertical bars represent mean ± standard error. The capital letters represent statistical significance on the analyzed day (Day 35 to 45) between different LED spectra

Overall, in most treatments, the antioxidant activities of the plant extracts were found to be slightly decreased over the analyzed period, as shown in Fig. 6, except for the BW treatment, which contributed an increase in the antioxidant activity at Day 45. Lettuce treated with the BW treatment possessed the highest antioxidant activity, followed by the control, GW, and RW, which resulted in less than 50% DPPH scavenging activity for all samples with measurement taken from Day 35 to Day 45. At the final day, the plant treated with the BW was found to have the highest antioxidant activity (86.8 ± 0.8% DPPH scavenging activity), which was 2.3-fold higher than that treated with the RW (37.5 ± 0.7% DPPH scavenging activity). The methanolic extract of lettuce grown under pure white LEDs possessed the DPPH scavenging activity of 62.1 ± 1%, while that of 45.3 ± 0.9% was obtained from the GW condition.

### Antioxidant enzymes

**Fig. 7:**
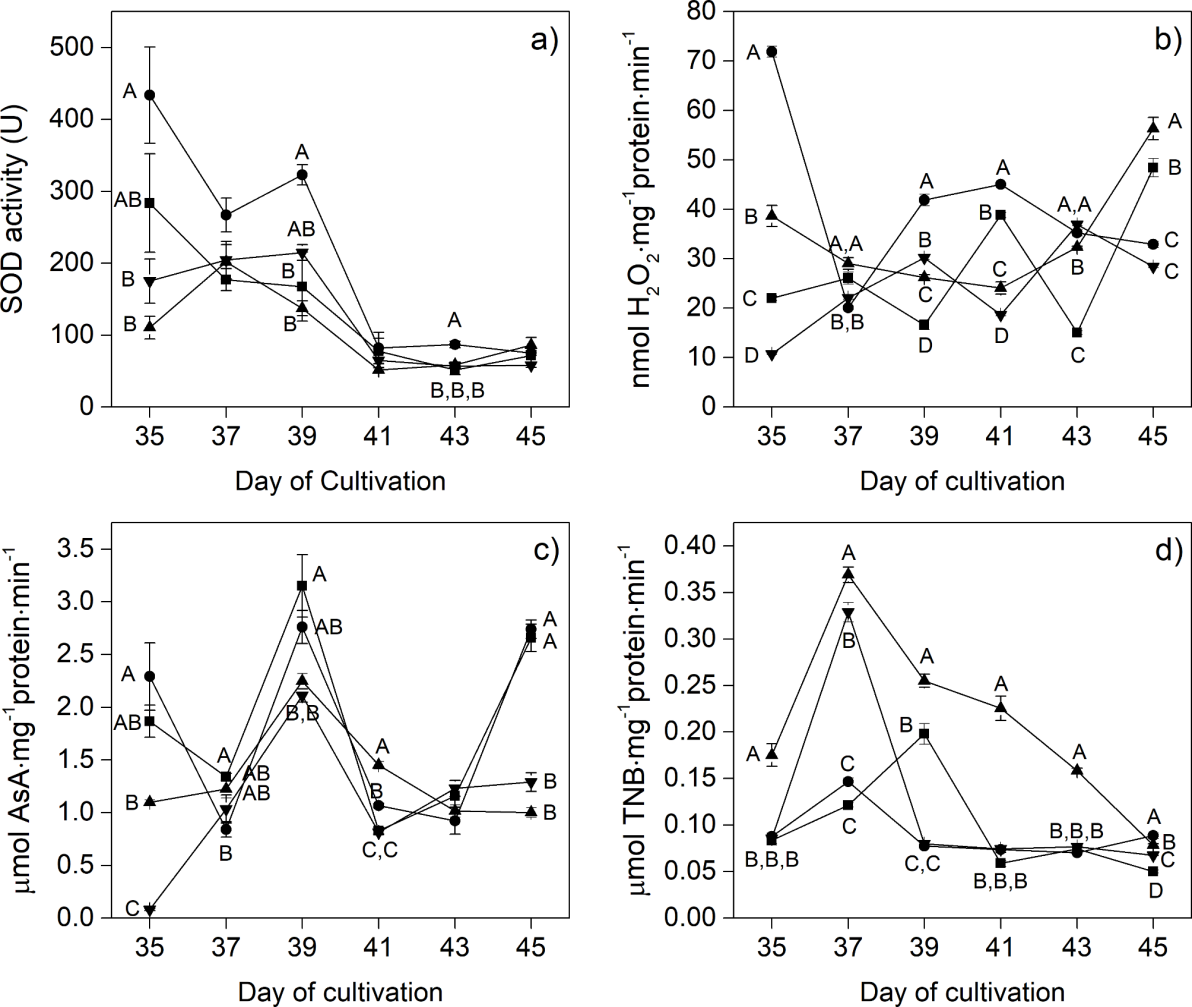
Antioxidant enzyme activities including (**a**) SOD, (**b**) CAT, (**c**) APX and (**d**) GR of red lettuce grown under different light spectrums including W (white, ■), BW (blue supplemented with white, λ_peak_ 442 nm, ●), RW (red supplemented with white, λ_peak_ 630 nm, ▲) and GW (green supplemented with white, λ_peak_ 517 nm, ▼). Vertical bars represent mean ± standard error. The capital letters represent statistical significance on analyzed day (Day 35 to 45) between different LED spectra. The absence of a letter label indicates no statistical significance between different LED spectra

Specific antioxidant enzyme activities can indicate the plants’ detoxification process in response to unfavorable conditions. Although low intensity (ca. 105 ± 10 µmol⋅m^-2^⋅s^-1^) normally did not induce any damages in plants, different spectra and/or long photoperiod (continuous light; 24 h photoperiod) may lead to plant stress [54]. Figure 7 shows that the antioxidant enzymes in the lettuce were influenced differently by the different LED spectra. Notably, the SOD activities in plants grown under all light treatments decreased gradually with time of cultivation, reaching lower than 100 units of SOD by Day 45 (Fig. 7a). At Day 35, lettuce grown under the BW LEDs exhibited a higher SOD activity (430 ± 67 Unit) than those grown under the white treatment (280 ± 68 Unit). The CAT activity, as demonstrated in Fig. 7b, was also affected by the light spectra. The profiles of CAT activity of lettuce from all treatments were found to fluctuate over the period of cultivation. The CAT activity of lettuce subjected to the BW treatment substantially reduced from Day 35 to Day 37, while those obtained from the other treatments showed small changes during this period. However, at the final stage of the experiment, the CAT activity of plants obtained from the RW treatment was found to be highest (56.3 ± 2 nmol H_2_O_2_⋅mg^-1^ protein⋅min^-1^), followed by those harvested from the white, BW, and GW. The changes in the APX activity of lettuce grown under different light spectra showed a similar trend (Fig. 7c); all treatments revealed their peaks at Day 39 of cultivation. By Day 45, the plants with highest activities of 2.7 ± 0.1 and 2.6 ± 0.1 µmol AsA⋅mg^-1^ protein⋅min^-1^ were observed from treatments by the BW and the control, respectively, followed by the RW and GW. In addition, the GR which is needed to regenerate active forms of antioxidants, was clearly influenced by the different wavelengths of light. According to Fig. 7d, all treatments except the control provided the highest GR activity for lettuce on Day 37, especially the RW (0.37 ± 0.01 µmol TNB⋅mg^-1^ protein⋅min^-1^). Subsequently, the GR activities were reduced in response to these treatments.

## Discussion

### Effect of light quality on fundamental properties of red lettuce

**Fig. 8:**
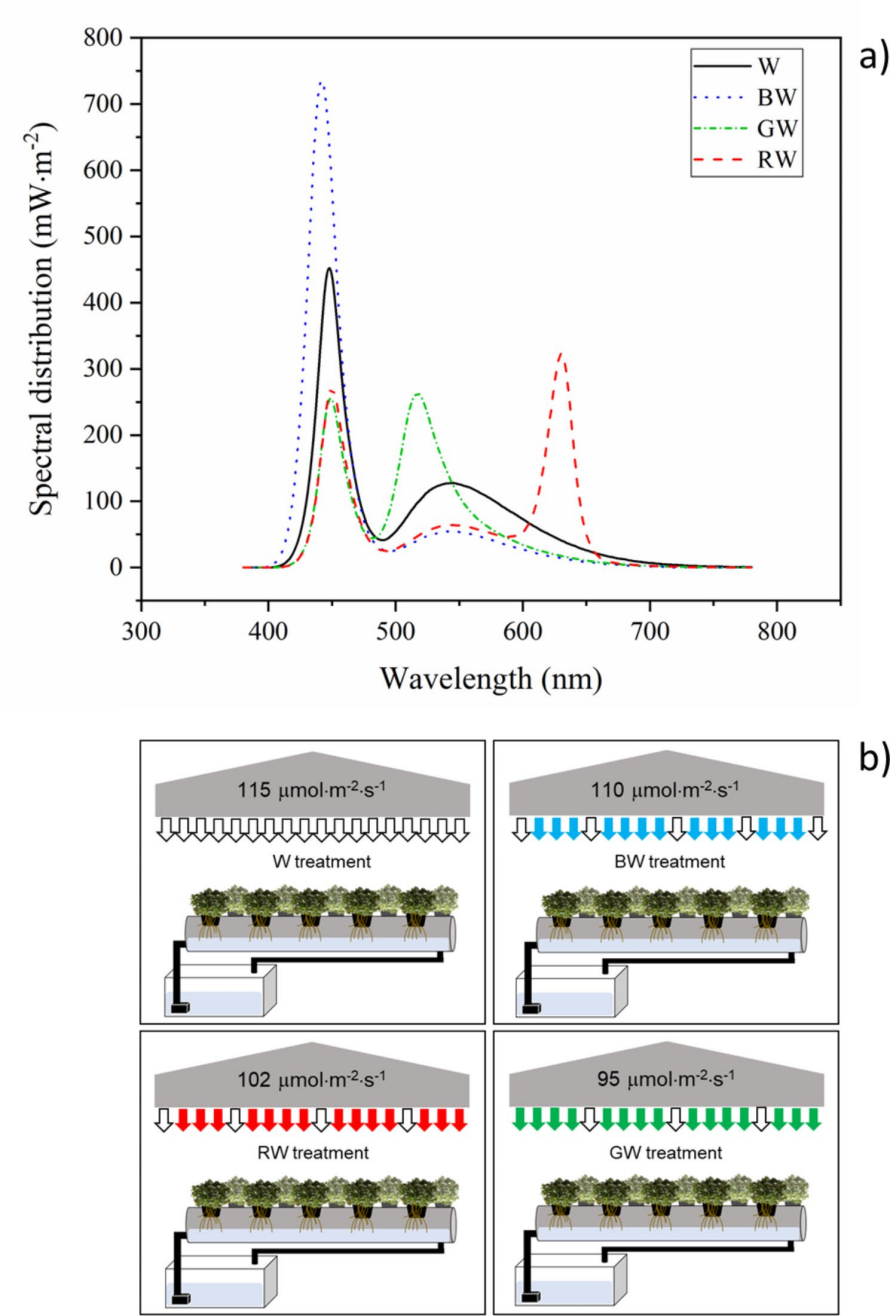
Experimental plant-setup with (**a**) different spectral distribution of LED spectra and (**b**) schematic diagram of growing light conditions in the study

Lettuce mass productivity, which is a fundamental property of vegetables, was found not to be significantly influenced by the changes in LED spectra examined in this study. Green LEDs alone were previously reported to be ineffective at producing lettuce with high productivity and good morphology [55]. However, when supplemented with a small amount of white LEDs, as in this study, no such negative results were observed. Son et al. [22] and others [56, 57] have reported a similar pattern, which supports the idea that white LEDs could promote mass production. Therefore, white supplementation in combination with various wavelengths (including blue, green, red and other wavelengths) may be deemed more suitable for boosting the overall biomass of lettuce than a monowavelength [44]. This could be one of the reasons that the current study did not observe differences in mass productivity. Another reason contributing to this trend could be the spectral distributions of all LED treatments, which provide some light in the blue wavelength range (400–500 nm), as shown in Fig. 8a. This may imply that favorable mass productivity could be due to the presence of light in this particular wavelength range, as blue light was found to induce stomatal opening, which leads to an increase in photosynthesis efficiency [58, 59]. Contrary to this, although green with high intensity was suggested to promote plant growth [33–35], the current study used this spectrum with a low intensity of ca. 105 ± 10 µmol⋅m^-2^⋅s^-1^. Thus, no mass productivity differences among treatments were caused by these mechanisms. The differences of plant responses between this study and some previous reports could be attributed to the different plant cultivar and different experimental conditions [10, 60]. Changes in the dietary fiber content displayed a similar trend to the changes in mass productivity. The results showed that the hemicellulose and cellulose contents of red lettuce were also not significantly different for all mixed-spectrum treatments. Generally, biomass production and dietary fiber content of vegetables strongly relate to each other [44], so it is not surprising that an effect of the light spectrum on the dietary fiber content was not observed in this study.

Leaf color is the first visual appearance that consumers consider when making a purchase decision. The current study also investigated the effect of continuous LED light with different spectra on the color of red lettuce leaves under controlled soilless cultivation. The changes in leaf colors observed to be influenced by the quality of artificial light, as shown in Fig. 1. The figure depicts that lettuce grown under the BW treatment possessed a higher amount of dark red in the leaves when compared to that under the control. In addition, the RGB profile was analyzed using ImageJ software [61] to estimate the proportions of red, green, and blue in images of the lettuce (200 × 150 pixels), as shown in Figs. S1 and S2 (Supplementary data). Fig. S2 confirms that the BW treatment provided lettuce leaves with comparable red and green intensities, while others resulted in lettuce with a predominantly green color. Obviously, the leaves under the BW treatment exhibited an obvious dark red or brown pigment, which was considered to be influenced by the combination of red pigment from anthocyanin, especially cyanidin and green pigment from chlorophyll molecules [44]. Previous works suggested that blue wavelengths could regulate the flavonoid pathway, which plays an important role in anthocyanin synthesis [45, 62]. Therefore, the higher redness of lettuce grown under the BW spectrum may imply that this treatment could provide the plant with a higher anthocyanin content than the others, contributing to greater health-promoting properties [63].

### Effect of light quality on phenolic compound accumulation of red lettuce

Different red pigment accumulations in lettuce leaves were observed in different LED spectrum treatments. To investigate the effect of LED spectra on this property, anthocyanin content was estimated from cyanidin-3-glucoside accumulation, which is a major component of the anthocyanin group in red lettuce. The HPLC results confirmed that cyanidin was accumulated in lettuce leaves grown under the BW treatment, with a 1.3-fold increase compared to the control at Day 45 (Fig. 3a). Previous studies also supported the conclusion that blue light influences the accumulation of anthocyanin in different varieties of lettuce, such as ‘Red Cross’ [15], ‘Banchu Red Fire’ [20], ‘Outredgeous’ [64], and ‘Cherokee’ [65]. This enhancement in cyanidin production may be attributed to the blue spectrum activating key enzymes, such as CHS (chalcone synthase) and DFR (dihydroflavonol-4-reductase) through cyptochrome (a blue/UV-A light photoreceptor) in the biosynthetic pathways of flavonoid/phenolic metabolites [66]. Apart from the increase in cyanidin accumulation, among eight detected phenolic compounds, chlorogenic acid showed high accumulation over the measured period in lettuce grown under the BW spectrum, as shown in Fig. 3c; it was more than 3-fold higher than that obtained from the RW treatment, which showed low accumulation at the 45th day. Moreover, most phenolic compounds were observed in high concentrations under the BW treatment for most periods of the measurements (Fig. 3). Heo et al. [46] and Landi et al. [67] suggested that the increase in the amount of most phenolics under the BW treatments could be driven by an increase in phenylalanine ammonia-lyase (PAL) activity (a key enzyme in the phenylpropanoid pathway) under light in the blue wavelength range. Furthermore, several studies have reported potential dependence of red wavelengths in promoting the accumulation of phenolic compounds. For example, Li and Kubota [15] and Samuoliene et al. [17] observed increases in phenolics concentrations by 28.5% in red baby lettuce and 6% in green lettuce, respectively, as exposed to red radiation. However, in this study, the RW treatment seems to be the least effective in stimulating phenolics accumulation. As a result, the summation of the detected phenolic compounds (mg phenolics per g DW) was high under the BW, followed by the control, GW, and RW treatments, respectively (Fig. 4a).

The summation of the productivity of the eight phenolic compounds (Fig. 4b), reported in the unit of content per unit planting area (m^2^) per growth period (days), showed a gradual increase with respect to time measurement for all treatments. Especially, the BW and the control were found to be the treatments that provided lettuce with the highest summation of these antioxidants over the planting period, followed by the GW and RW. While white light in the control treatment could promote an improvement in the fresh weight of lettuce, the BW was more efficient in terms of total phenolics productivity (mg⋅m^-2^⋅day^-1^), which combines basic phenolics biosynthesis with mass productivity. This confirms that the phenolics biosynthesis pathway was influenced by the blue part of the spectrum, and it suggests that the increase in mass productivity under the control treatment exceeded the rate of phenolics synthesis [68, 69]. These results have been supported by the Folin-Ciocalteu method (Fig. 5), which indicates that the blue radiation could improve the total phenolics accumulation better than other wavelengths. The GW treatment, on the other hand, was found to provide a lower phenolics accumulation. This finding aligns with previous studies [70, 71], which suggested that the green spectrum played a role in inhibiting the activation of cryptochrome, resulting in a decrease in flavonoids and anthocyanins. Phenolic compounds have been realized to either function as direct antioxidants or to increase the production of other antioxidant compounds in the human body [72]. As a result, healthy compounds contained in lettuce, as measured from the total quantity of phenolics accumulation, are shown to be influenced directly by the light quality. However, when considering total phenolic production (Fig. 5a), it increased from Day 43 to Day 45 in the BW treatment, while the sum of the eight detected phenolic compounds (Fig. 4a) did not. In addition, this study found that the results of antioxidant activity for all treatments (Fig. 6) provided a more positive correlation with their total phenolic production (Folin-Ciocalteu method) than the summation of eight detected phenolic compounds (analyzed by HPLC). For instance, in the case of BW, total phenolic production and antioxidant activity show a high correlation coefficient of 0.90, while that between the summation of eight detected phenolic compounds and antioxidant activity was 0.63. This may be because red lettuce produces significant phenolics other than those eight compounds. Thus, subsequent research should take into account additional compounds beyond the aforementioned eight.

### Effect of light quality on antioxidant activity of red lettuce

The quality of the antioxidant defense system was evaluated via the percentage of DPPH scavenging. The results demonstrated that the extract of plants grown under the BW treatment possessed the highest antioxidant activity, whereas the RW treatment was observed to be the worst in this regard. This contradicts previous research [73–75] in which red spectra showed a tendency to provide greater antioxidant activity than green. Previous reports typically revealed a positive correlation between antioxidants accumulation and total antioxidant activity [76], as did this study that found a high level of antioxidant activity in lettuce extracts rich in phenolic compounds (phenolic acids, and flavonoids). In addition, Cai et al. [77] reported that an increase in antioxidant activity directly depends on an accumulation of the phenolic hydroxyl group. This may imply that the BW not only promoted the synthesis of antioxidants but also that the compounds produced were those with superior free radical scavenging activity. In other words, the treatment provided positive effects in this regard, not only quantitatively but also qualitatively.

### Effect of light quality on antioxidant enzyme activities of red lettuce

Light of varying qualities and intensities has been shown to create unpleasant environments for plants, which can trigger the production of both non-enzymatic and enzymatic antioxidants in response [78]. It is important to note that the units of antioxidant activities depend on the standard method used for their measurement, so the four enzyme activities measured in this study were reported in different units. This makes it difficult to compare activities across different enzymes, however, high enzyme activities (for any of these species) implies that plants are fighting against a high amount of reactive oxygen species (ROS) from unsuitable growing conditions [79]. This research indicated that changes in antioxidant enzyme activities involved in the defense system were induced by light quality. The SOD and GR activities were found to drop, while the CAT and APX activities fluctuated over the period of plant growth. Notably, it appears that the varying LED spectra applied in the experimental setting possessed no significant impact on inducing stress in plants, as evidenced by the observed positive effect on plant growth and the fluctuating activities of all the enzymes. The reduction of SOD activity may indicate that different spectra of light did not stimulate SOD, which is the first line of defense against the excess ROS [39]. Similarly, the activities of GR in lettuce from all treatments were found to peak on Day 37, which may be caused by suppression of CAT and APX on that day [80, 81]. It is interesting to note that the BW treatment showed a tendency for high SOD and CAT activities, while the RW treatment showed high GR activity. To clarify the effect of light quality on antioxidant enzyme activities, it is necessary to quantify ROS (O_2_^•-^, H_2_O_2_), ascorbic acid, and glutathione in further studies. Moreover, although the RW treatment presented relatively outstanding GR activity for lettuce, its antioxidant accumulations (total phenolic content and antioxidant compounds as analyzed by HPLC) were clearly lower than those observed in other treatments. Changes in the GR activity as a result of the RW treatment may be attributed to an uncomfortable condition caused by a 24-hour photoperiod of continuous light exposure, in which the plant’s enzymatic defense system could efficiently function to delay or eliminate oxidative compounds. Thus, the biosynthesis of antioxidants, which is the second line of defense, was not significantly promoted under RW LED regulation.

## Conclusion

The current study demonstrated the impact of mixed LED wavelengths on lettuce growth and its antioxidant defense system (enzymatic and non-enzymatic). It was illustrated that the four LED wavelengths greatly affected chemical adaption (antioxidant defense system), but not fundamental properties (biomass and dietary fiber) of soilless-cultivated red lettuce. The biomass and fiber accumulation of the lettuce may have been maintained partially due to the small supplementation of white LED in the other mono-wavelengths, which helped maintain the plant growth. The use of blue supplemented with white could efficiently increase a number of health-promoting compounds. Although monochromatic green LED generally produces a negative impact on plant development [32, 82], the GW treatment provided a higher phenolics concentration than the RW treatment. Light adjustment can be a practical strategy to manipulate plant quality. Consequently, it could be implied that not only suitable fertilizer nutrients but together with proper light quality can increase health-promoting compounds even more. To understand the antioxidant responses at the molecular level, the effect of light quality, along with other agricultural factors, on key genes related to the antioxidant defense system should be further studied.

## Materials and methods

### Plantation setup and plant material

Coated red lettuce seeds (Enza Zaden Brand) were imported from the Netherlands via the SP hydroponic company, Thailand and were germinated in 1 × 1 × 1 cm^3^ sponges. Seeds were watered twice a day, and kept in a laboratory with an average temperature of 25 °C, 70–80% relative humidity, and grown under white light for 10 days. Healthy sprouts were transferred to a controlled recirculating hydroponic system (Fig. 8b). A modified Huett’s nutrient solution (pH 6.8 and EC 1152 µS⋅cm^-1^) [83] comprising of (mM) nitrogen (N) 8.29, potassium (K) 2.56, calcium (Ca) 1.75, magnesium (Mg) 0.41, phosphorus (P) 0.71, sulfur (S) 0.81, iron (Fe) 0.045, manganese (Mn) 0.004, boron (B) 0.019, zinc (Zn) 0.0023, copper (Cu) 0.00047, and molybdenum (Mo) 0.0001 was applied and adjusted once a week. An experimental room containing four different light treatments was maintained at 22-25 °C throughout the experiment using air conditioners. Experiments for each treatment were located in a reflective grow tent, which included a 1 m^2^ planting area (25 plants per m^2^ planting density), an LED panel (1.56 m^2^) and a fertilizer tank. The LED panel was made from patching LED strips (5 m⋅strip^-1^, 12 V, 14.4 W⋅m^-1^, 60 LEDs⋅m^-1^) onto a polypropylene sheet with different ranges of wavelengths. In experiments using color LED strips, white LED chips (ca. 20–30% of the total chips) were added to provide a total photosynthetic photon flux density (PPFD) of 105 ± 10 µmol⋅m^-2^⋅s^-1^ (24 h photoperiod), measured using a hand-held spectrometer (MK350N Premium, UPRtek Corp., Taiwan) which matched the PPFD of the white light experiment (Fig. 8a). The duration of the photoperiod was 24 h. In addition, white LEDs create a broad spectrum, which provides more efficiency in photosynthesis than a narrow one [57]. The maximum energy level of each light source was 452.5 mW⋅m^-2^ for the W treatment, 736 mW⋅m^-2^ for the BW treatment, 263.1 mW⋅m^-2^ for the GW treatment, and 323 mW⋅m^-2^ for the RW treatment. Since the LED chips were not fully monochromatic and some white light was used to normalize the PPFD, the experiments performed with color LEDs strongly supplemented a particular part of the light spectrum while reducing intensity in other wavelengths, not entirely removing them. With the addition of white LEDs, a hand-held spectrometer was used to measure the wavelength of the obtained mixed lights. This is to ensure that the spectral outputs remained within the ranges of the intended light colors, which were 400–500 nm, 500–600 nm, and 600–700 nm for blue, green, and red, respectively. Table 1 demonstrates the correlation between the ratios of different color LEDs and the resulting peak wavelengths, which explains this setting. The light panels were located 50 cm above the planting area. The modified Huett’s nutrient solution was pumped into planting channels and checked weekly using Inductively Coupled Plasma Optical Emission Spectroscopy (710 ICP-OES, Agilent Technologies, USA) to maintain the nutrient levels. At the same growing position, the plants were harvested from each treatment on Days 35, 37, 39, 41, 43, and 45 after seed germination (Fig. S3), when they had reached sufficient mass for analysis. The experiment was conducted in triplicate for all treatments.

**Table 1:** Spectral information of light treatments including white as a control (W), blue supplemented with white (BW), green supplemented with white (GW) and red supplemented with white (RW).

Treatment	Spectral distribution			Peak wavelength (nm)	Total photosynthetic photon flux density (PPFD) (µmol⋅m^-2^⋅s^-1^)
	%Blue	%Green	%Red		
W	50	38	12	448	115
BW	81	15	4	442	110
GW	38	57	6	517	95
RW	35	24	41	630	102

### Measurement of mass productivity and dietary fiber content

At Day 45, at least ten fresh lettuce plants at the same position in the planting areas were trimmed to remove the roots and weighed for calculating mass productivity in the units of gram of fresh weight (FW) per unit of planting area per time of cultivation (g⋅m^-2^⋅day^-1^). Dietary fiber content (cellulose and hemicellulose content) was estimated following a modified Van Soest analysis [84, 85]. Briefly, 200 g of fresh leaves at Day 45 were cut into small pieces and digested with 500 mL of 96% ethanol for 20 min at 85 °C [86]. The digested leaves from each treatment were processed by a kitchen blender for 15 min, after which the solid fraction was separated from the liquid part using a filter cloth. The soluble portion of the solid was then extracted with 70% ethanol for 20 min. After removing ethanol by filtration, the solid residue was thoroughly washed with 96% ethanol followed by acetone. After washing, the samples were dried overnight in an oven at 40 °C; the remaining solid was the cell wall material.

An acid-detergent solution (AD) was prepared for the determination of cellulose content. Briefly, 20 g of cetyltrimethylammonium bromide (CTAB) was dissolved in 1 L of 0.5 M sulfuric acid. 0.5 g of the cell wall material was added to a mixture of 100 mL of AD solution and 2 mL of decahydronaphthalene. All ingredients were refluxed at 210 °C for 10 min. To avoid foaming, the temperature was reduced to 185 °C and this temperature was maintained for 1 h. The mixture was then filtered through a filter paper (Whatman No. 1), then thoroughly washed with hot water (95 °C) followed by pure acetone. The solid residue was subsequently dried overnight in an oven at 100 °C. The mass of the dry residue was defined as m_ADF_ which was further used in Eq. 1 for the calculation of the cellulose content.

A neutral-detergent solution (ND) was prepared for the determination of hemicellulose content. The solution was prepared by mixing 18.61 g of disodium ethylene diamine tetraacetate (EDTA), 6.81 g of sodium borate decahydrate, 30 g of sodium lauryl sulfate, 10 mL of 2-ethoxyethanol, and 4.56 g of disodium hydrogen phosphate in 1 L of heated deionized water. The solution was heated until all chemicals dissolved, then 0.5 g of the cell wall material (m_CWM_) was added to a mixture of 100 mL of ND solution, 2 mL of decahydronaphthalene and 0.5 g of sodium sulfite. All steps were conducted following the previous cellulose determination procedure. The solid part was dried overnight at 100 °C in an oven, and the weight of the residue was defined as m_NDF_. The hemicellulose content was then calculated from Eq. 2, which assumes that lettuce contains 95% water content [87, 88].

1 $$\begin{array}{l}Cellulose\,yield\left( {mg \cdot {g^{ - 1}}FW} \right) = \\\left( {\frac{{{m_{ADF}}}}{{{m_{CWM}}}}} \right) \times \left( {\frac{{5\,g\,DW}}{{100\,g\,FW}}} \right) \times \left( {\frac{{1000\,mg}}{{1\,g}}} \right)\end{array}$$

2 $$\begin{array}{l}Hemicellulose\,yield\,\left( {mg \cdot {g^{ - 1}}FW} \right) = \\\left( {\frac{{{m_{NDF}} - {m_{ADF}}}}{{{m_{CWM}}}}} \right) \times \left( {\frac{{5\,g\,DW}}{{100\,g\,FW}}} \right) \times \left( {\frac{{1000\,mg}}{{1\,g}}} \right)\end{array}$$

### Plant extraction and determination of phenolics accumulation

Fresh leaves at the same position for each treatment were harvested and freeze-dried for 48 h. Aqueous methanol (MeOH) (12.5 mL of 80% v/v) was added into a centrifuge tube with 0.5 g dry weight (DW) of sample and sonicated for 30 min at 35 °C. This was followed by centrifugation at 12,000×g for 10 min. The supernatant part was filtered using a 0.45 μm nylon filter and collected in an evaporating flask. The solid residue was extracted twice with the same amount of solvent. The solvent was eliminated from the flask with all the supernatant with a rotary evaporator (40 °C for 20 min), then dried in a vacuum oven (25 °C, overnight). The obtained crude was dissolved in 15 mL of 80% MeOH, filtered, and stored at -18 °C until required for analysis [89].

### Measurement of total phenolic content

The modified Folin-Ciocalteu method used by Khandaker et al. [90] was applied to estimate the total phenolic content in the extract of red lettuce. 125 µL of plant extract was reacted with 2.5 mL of Folin–Ciocalteu solution (a ratio of reagent: distilled water of 1:4) in an amber bottle. After 3 min, 2.5 mL of 10% aqueous sodium carbonate was added, and then the mixture was kept in the dark for 1 h at room temperature. The absorbance of the mixture was measured at 760 nm using a spectrophotometer (Cary 100, Agilent Technologies, USA). The results are reported in units of gallic acid equivalent (GAE).

### Determination of antioxidant activity

The 2,2-Diphenyl-1-picrylhydrazyl (DPPH) free-radical scavenging assay from Khandaker et al. [90] was modified to determine the antioxidant capacity of plant extract. A sample of 200 µL of diluted extract (0–25 mg⋅mL^-1^) was mixed with 1 mL of DI water and 4 mL of 0.1 mM DPPH in 80% MeOH in an amber bottle for 30 min in dark conditions. The absorbance of the mixture was measured at 517 nm using a spectrophotometer (Cary 100, Agilent Technologies, USA). The antioxidant activity of a 10 mg DW⋅mL^-1^ methanolic lettuce extract was determined via the percentage of DPPH scavenging activity, which is calculated following Eq. 3, where A_control_ is the absorbance of the mixture without lettuce extract and A_sample_ is the absorbance of the mixture with lettuce extract.

3 $$\% \,DPPH\,scavenged = \left( {\frac{{{A_{control}} - {A_{sample}}}}{{{A_{control}}}}} \right) \times 100$$

### Measurement of specific phenolic compounds using high performance liquid chromatography (HPLC)

The quantities of the main phenolic compounds of red lettuce, including cyanidin-3-glucoside, gallic acid, chlorogenic acid, vanillic acid, caffeic acid, quercetin-3-O-glucopyranoside, quercitrin, and luteolin were determined using a C18 HPLC column (250 × 4.6 mm, 5 μm particle size). Samples were analyzed at 25 °C using a 15 µL injection volume, 0.5 mL⋅min^-1^ for the mobile phase flow rate, and the mobile phases were composed of 2% (v/v) acetic acid aqueous solution (A) and pure acetonitrile (B). The gradient of the mobile phases was 0–7 min 90% A, 7–15 min 85% A, 15–32 min 45% A, 32–38 min 25% A and 38–60 min 90% A, as suggested by Marcussi et al. [91].

### Determination of antioxidant enzymes

For enzyme extraction, 1 g of young fresh leaves were ground with liquid nitrogen in a cooled mortar and pestle. The sample powder was homogenized in a cool 25-mL tube with 1.5 mL of 0.1 M phosphate buffer, pH 7.5 with 0.5 mM EDTA for SOD, CAT, GR and total protein analysis [92] and with 2 mL of phosphate buffer 50mM, pH 7.0 with 0.1 mM EDTA, 5% polyvinylpyrrolidone (PVP-40) and 1 mM ascorbic acid for APX analysis [93]. The tube was centrifuged at 12,000×g for 15 min. The supernatant was removed and collected in a small amber bottle. The solid residue was extracted again with the same amount and type of solvent for each analysis. All procedures were conducted at 4 °C. The combined supernatant was filtered using a 0.45 μm filter and antioxidant enzyme activities were estimated within a few hours. Since plant enzymatic tests are unstable and sensitive, three plants from each treatment were harvested for independent measurements. Each extract sample from each plant was also analyzed three times independently. This means that each treatment had nine antioxidant enzyme activity measurements.

Superoxide dismutase (SOD) activity was measured using the modified nitroblue tetrazolium (NBT) assay [92, 94]. In a reaction cuvette, a 2070 µL mixture containing a 40 µL enzyme sample, 50 mM buffer (pH 7.8) with 2 mM EDTA, 9.66 mM L-methionine, 50 µM NBT, 0.024% Triton-X100, and 9.66 µM riboflavin (added last) was illuminated by a 24-watt fluorescent light for 5 min. The absorbance at 560 nm was measured using a spectrophotometer (Cary 100, Agilent Technologies, USA) for the calculation of the SOD activity. A non-illuminated cuvette was used as a blank. The SOD activity is calculated following the formula (Eq. 4) of Zhang et al. [95].

4 $$\begin{array}{l}SOD\,activity\,\left( {U;unit} \right) = \\\left( {\frac{{{A_0} - {A_1}}}{{{A_1}}}} \right) \times \left( {\frac{{{V_{system}}}}{{{V_{sample}}}}} \right) \times DilutionFactor\end{array}$$

where A_0_ is the absorbance of the mixture without enzyme and A_1_ is the absorbance of the sample. The volumes of the total mixed solution and enzyme sample are V_system_ and V_sample_, respectively.

Catalase (CAT) activity was measured following Elavarthi and Martin [92] and Aebi [96]. A mixture was prepared from a 200 µL enzyme sample, 2000 µL of 50 mM buffer (pH 7) and 1000 µL of 30 mM H_2_O_2_ (added last). The absorbance at 240 nm was measured using a spectrophotometer (Cary 100, Agilent Technologies, USA) and recorded immediately for a period of 3 min. The CAT activity was calculated as the decomposition of H_2_O_2_ per minute per milligram of protein (extinction coefficient 40 mM^-1^⋅cm^-1^).

Ascorbate Peroxidase (APX) activity was assayed using the methods of Mizuno et al. [93] and Nakano and Asada [97] with some modifications. The reaction consisted of 250 µL enzyme sample, 2600 µL phosphate buffer (50 mM), 200 µL ascorbate (300 mM), and lastly 200 µL H_2_O_2_ (30 mM) were added to start the reaction. The decrease in the absorbance at 300 nm was measured immediately for a period of 3 min. The activity was defined in terms of the amount of reduced ascorbate (AsA) per minute per milligram of protein (extinction coefficient 0.74 mM^-1^⋅cm^-1^).

Glutathione Reductase (GR) activity was estimated according to Elavarthi and Martin [92]. A 200 µL enzyme sample was added to a cuvette along with 50 mM buffer (pH 7.8) with 2 mM EDTA, 0.09 mM NADPH, 0.61 mM DTNB, and 0.82 mM GSSG (added last) in a total of 2450 µL solution. The increase in absorbance at 412 nm was measured immediately for a period of 3 min. GR activity was calculated using an extinction coefficient of 14.15 mM^-1^⋅cm^-1^ and expressed as the amount of TNB per minute per milligram of protein.

Total protein content was determined following the Bradford assay [98], using BSA (Bovine Serum Albumin) as a standard compound.

### Statistical analysis

Experiments were conducted in triplicate for all treatments. On the day of measurement, three plants per treatment were harvested to determine all antioxidant properties, including total phenolic content, specific phenolic compounds accumulation, antioxidant activity, and antioxidant enzyme activities. Plant extracts from each treatment were also measured three times. For mass productivity and dietary fiber content determination, at least ten mature plants were harvested per treatment. The results were reported as the mean ± standard error. Statistical analysis was carried out by one-way analyses of variance (ANOVA) and Tukey pairwise comparisons at a significance level of 0.05 (Minitab 19).

## Electronic supplementary material

Below is the link to the electronic supplementary material.

Supplementary Material 1

## Data Availability

All data relevant to the results and analysis in this study are included in this article and its supplementary materials.
