# Supplementary material for: Effect of mixed light emitting diode spectrum on antioxidants content and antioxidant activity of red lettuce grown in a closed soilless system
Source: BMC Plant Biol. 2023 Jul 6;23:351. doi: 10.1186/s12870-023-04364-y (PMC10324264; doi:10.1186/s12870-023-04364-y)
Supplement: Supplementary file 1 — Supplementary Material 1 [file 12870_2023_4364_MOESM1_ESM.docx]

Effect of mixed light emitting diode spectrum on antioxidants content and antioxidant activity of red lettuce grown in a closed soilless system

Sopanat Sawatdee^1^, Teeraya Jarunglumlert^2^, Prasert Pavasant^3^, Yasuko Sakihama^4^, Adrian E. Flood^1,*^, Chattip Prommuak^5,*^

**Additional data**


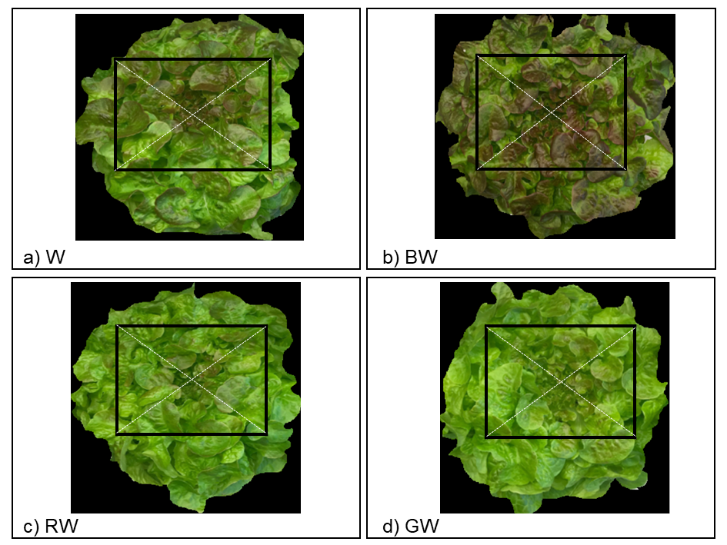


**Fig. S1** Rectangular area selected for RGB profile analysis of each 45-day lettuce image. The area is 200 width × 150 height pixels and diagonal lines pass through the center of image.





**Fig. S2** RGB profiles of selected area of lettuce images from different LED spectrum treatments including a) white (control), b) BW (blue supplemented with white, λ_peak_ 442 nm), c) RW (red supplemented with white, λ_peak_ 630 nm) and d) GW (green supplemented with white, λ_peak_ 517 nm). Solid, dashed and dotted lines represent red, green and blue intensity, respectively.


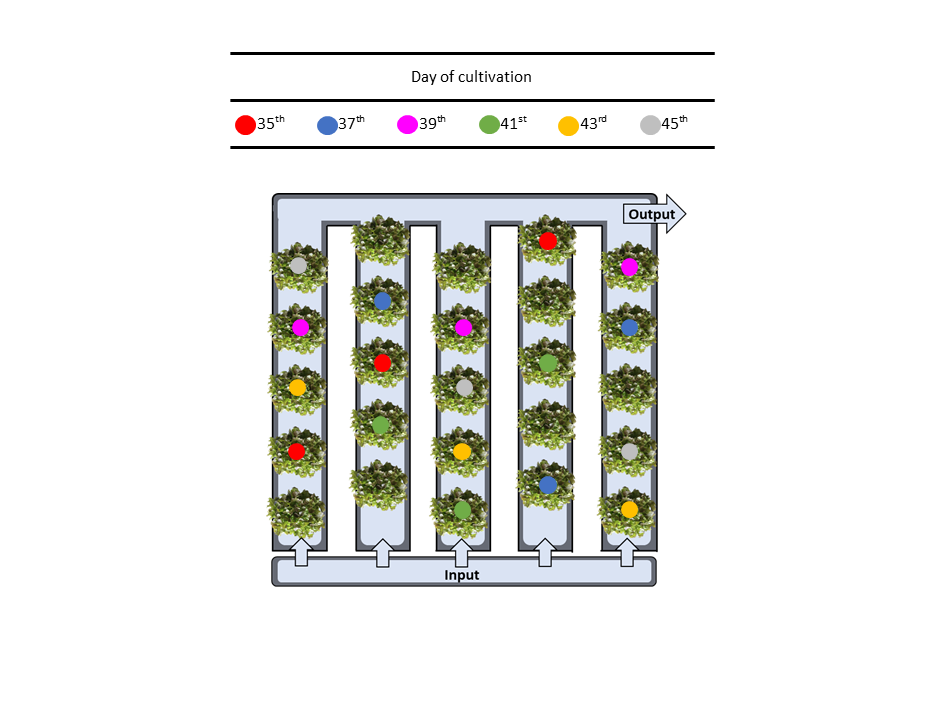


**Fig. S3** The position of harvested plants in the study. Three plants in each light treatment were sampled at the same position. For example, plants in red dot positions were harvested at Day 35.
